# CRISPR technologies for the control and study of malaria-transmitting anopheline mosquitoes

**DOI:** 10.1186/s13071-025-06905-w

**Published:** 2025-07-03

**Authors:** Andrea L. Smidler, Omar S. Akbari

**Affiliations:** School of Biological Sciences, Department of Cell and Developmental Biology, University of California, La Jolla, San Diego, CA 9209 USA

## Abstract

Malaria is one of the deadliest diseases on the planet, killing approximately 600,000 people annually, and is transmitted by the bite of an anopheline mosquito. Anophelines, and the diseases they transmit, have changed the course of history and the fate of nations, and their successful control promises to end the transmission of malaria. With the advent of clustered regularly interspaced short palindromic repeats (CRISPR)/CRISPR-associated protein 9 (Cas9) technologies, the study and control of these deadly pests have been revolutionized. As the release of genetically modified anophelines is being considered, here we outline the advances in CRISPR/Cas9 technologies and how they have revolutionized the study of anopheline basic biology and the development of innovative vector control strategies. We outline the major findings of CRISPR-based basic biological research into traits relevant for vector control including, but not limited to, olfaction, chemosensation, neurobiology, and reproduction. Further, we summarize the advancements in CRISPR-based innovative vector control strategies, such as the precision-guided sterile insect technique (pgSIT), inherited female elimination by genetically encoded nucleases to interrupt alleles (IFEGENIA), X-shredder, Y-linked editors, and gene drives. All in all, this review summarizes the basic biological and vector control research undertaken using CRISPR since its advent approximately a decade ago.

**Graphical Abstract:**

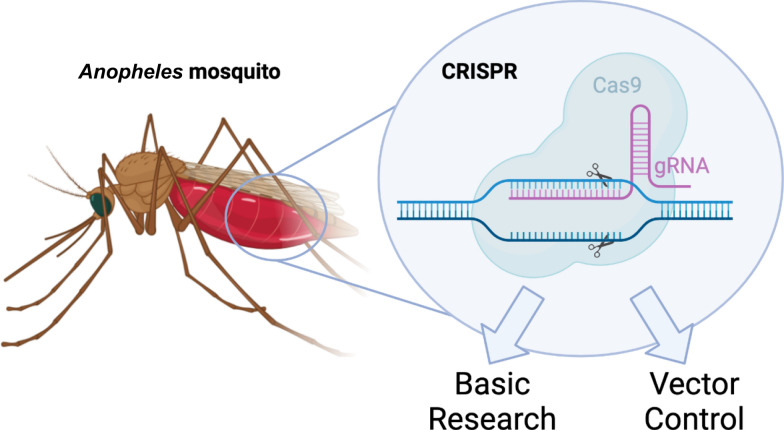

## Background

**Fig. 1:**
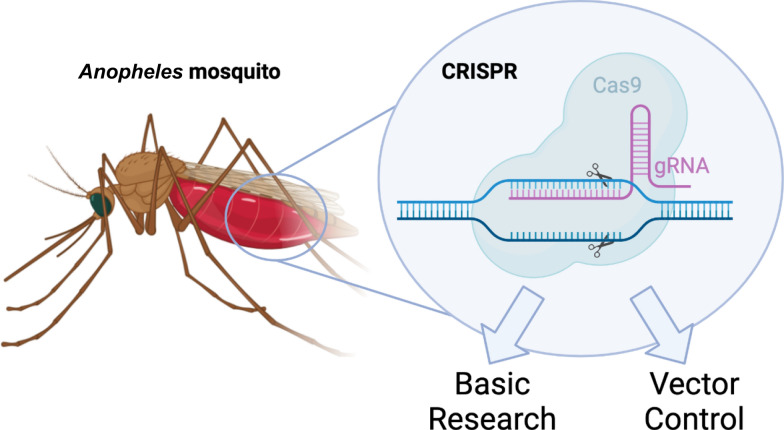
Clustered regularly interspaced short palindromic repeats (CRISPR) have been used to develop vector control tools as well as enable research into the basic biology of malarial *Anopheles* mosquitoes

Transmitted by the bite of anopheline mosquitoes, malaria not only kills over half a million people annually [1], but has also shaped the fundamentals of human genetics, the design of ancient civilizations, and the fate of modern nations. But with the advent of CRISPR genome engineering, the control and study of these disease-transmitting insects have been revolutionized, and disease eradication might finally be within our grasp. All thanks to programmable protein complexes so tiny that nearly 340,000 of them could be lined up end-to-end across the head of a pin. Herein, we discuss the CRISPR applications for the control and study of malaria-transmitting *Anopheles* mosquitoes, providing a brief contextualization of predecessor technologies, and a comparative discussion of similar technologies developed in non-anopheline Diptera but which are ripe for development in anophelines (Fig. 1).

## Lessons from the past and historic malaria control

Human genetics have been shaped by malaria, with traits such as sickle cell, G6PD, and Duffy conferring protection against infection [2, 3]. Malaria was endemic in much of the world before the mid-century and has had a notable impact on society since the beginning of recorded history [4]. In the Americas, it may have been brought over via the slave trade, and then became endemic through transmission by native anophelines. It turned the tide of the American Revolutionary War when it decimated the susceptible British troops in South Carolina but not the resistant American troops [5]. Malaria motivated the construction of the ancient Roman aqueducts, enabling wealthy Romans to construct their villas away from the lowland malaria-ridden swamps [6]. However, malaria is not endemic in these locales today. Malaria was eradicated from these regions by using the following ecologically destructive methods to control mosquitoes: removing wetlands, oil-slicking swamps, and applying copper acetoarsenite [7–9]. In Italy, the number of cases in some regions fell to zero by 1939 [7, 10]. During these campaigns, the endogenous mosquito population was nearly eliminated for decades, suppressed to such an extent that malaria transmission was interrupted. When coupled with improved living conditions and the distribution of quinine to treat lingering cases, malaria eradication was achieved in many regions. As a result, vector control interventions were stopped, and the native mosquito species recovered. Now, only rare local transmission occurs when malaria is imported, making these areas functionally malaria-free.

This history has important lessons for modern malaria control. Notably, vector control is a potent tool for the control of malaria transmission, and is considered essential to stop disease spread; however, ideally we will move forward by using more modern, ecologically friendly methods. The use of insecticides and bed nets forms the crux of malaria control campaigns, but evolving resistance is making their use less efficacious [11]. While some are hoping that the right mosquito intervention might even be a silver bullet [12], general consensus is that it will take a multi-pronged approach to eradicate malaria globally. Importantly, regardless of which tools are implemented, it is important to note that vector control interventions need not be permanent. Once the malaria parasite is eliminated, suppression interventions can be ceased, allowing native mosquito species to rebound—especially when imported cases are low. These examples provide tangible evidence as to the minimal ecological effect of eliminating malaria vectors from the environment. Importantly, the ecological impacts of malaria vector elimination in Southern Europe and North America are not a cautionary tale today^1^ providing a valuable piece of evidence that temporary mosquito eradication may not catalyze an ecological disaster.

## Effects of anopheline suppression and replacement

Modern genetic-based vector control tools rely on population suppression, the elimination of the vector, or population modification, replacing the disease vector with a population incapable of transmission, and do so in a highly species-specific manner. Many modern vector control methods rely on population suppression to disrupt disease transmission; eliminating the vector eliminates the disease. The most commonly known modern efforts to control mosquito transmitted diseases involves the suppression of *Aedes* in South America, Florida, and California [14, 15], while the nearly complete historic eradication of malaria in Southern Europe and North America is a prime example of successful disease eradication following anopheline suppression. These successes also demonstrate how suppression efforts need not be permanent but can achieve elimination by limiting imported cases and enduring long enough to interrupt transmission to zero. The native malaria vectors responsible for endemic and epidemic malaria can rebound over time and have done so in the malaria-free areas of Southern Europe and North America [16, 17], proving that eradication of mosquitoes in their native range need not be permanent and can still have successful outcomes. However, many mosquito disease vectors are invasive and have become established outside their native range. *Anopheles stephensi*, for example, is a native Asian malaria vector, but has recently invaded parts of Africa [18], while *Aedes aegypti,* originally an African mosquito, is now present in much of the temperate USA. Suppression programs targeting invasive species would likely focus on permanent eradication from non-native regions.

Even in their native distribution, malaria vectors are not considered keystone species, and their removal will likely have little impact on the local ecology. Many malaria mosquito larval habitats are most abundant in human-disturbed environments [19]. A study in South Africa showed that natural mosquito communities, outside of areas of intense human influence, have diverse species composition and minimal malaria vectors [20]. This finding is unsurprising since of the over 3500 mosquito species, only a small fraction vector disease [21]. In fact, five main species are thought to cause 95% of the malaria cases in Africa [22]. Therefore, even if a few native mosquito species are eradicated to eliminate malaria, thousands of other native mosquito species that pose little risk to human health can thrive, particularly in more biodiverse natural habitats. However, it is important to note that there is debate over how anthropophilic these species are, and thus how well they would fill the niche surrounding primarily human- and cattle-occupied villages.^2^

## Modern malaria control

Modern malaria control efforts focus heavily on controlling wild mosquito populations. With almost 600,000 cases annually, curtailing its continued spread is of utmost importance [23]. Treating or removing breeding sites helps control the larval aquatic stage [24], and medicinal treatment relies on the use of chloroquine or artemisinin-based therapies to treat cases. Insecticide-treated bed nets and indoor residual spraying are used continent-wide to control adult biting populations [11]. However, insecticide resistance is emerging at an alarming rate, thus making these control efforts increasingly ineffective, and urgently necessitating the development of novel vector control tools. Genetically modifying mosquitoes promises to open doors to novel, potentially more eco-friendly vector control options, and support for their release has gained traction [25–27].

This review aims to address the ways in which CRISPR has revolutionized the research and control of malarial anophelines. To provide context, background on the ways in which non-CRISPR technologies have shaped the field and laid the groundwork for today's advances is warranted, in addition to mentions of select advances made in drosophilid species.

## Alternative vector control tools—*Wolbachia* and incompatible insect technique-based control

Technologies such as incompatible insect technique (IIT) and release of insects carrying dominant lethal (RIDL), have been used to control *Aedes* mosquitoes to great effect [28, 29]. In IIT, male mosquitoes carrying the endosymbiotic bacteria* Wolbachia* are released, which when mated to a wild female without* Wolbachia* cause embryonic lethality through a mechanism called cytoplasmic incompatibility (CI). However, male-only populations must be released to prevent establishment of the maternally transmitted* Wolbachia* bacterium into the population. Release of rare* Wolbachia*-containing females may lead to failure to suppress the population and failure of the intervention. This has necessitated supplementation with radiation-based sterilization to sterilize surviving females to guarantee their elimination from the population [30]. Successful use of* Wolbachia* in IIT has been limited to *Aedes* populations of mosquitoes, as most anopheline species lack* Wolbachia* [31, 32]. Rare natural* Wolbachia* anopheline infections do not display CI [33], and synthetic trans infectious germline infections are notoriously difficult to establish [34, 35], albeit with some success noted in *A. stephensi *[36]. Somewhat similarly, RIDL or female-specific RIDL (FsRIDL) involves the release of fertile males with a chemically repressed lethal transgene, which when expressed in the progeny (in the absence of the chemical repressor) causes offspring lethality or incapacitation [37–39]. And while RIDL has been implemented in *A. stephensi*, it has yet to be tested in larger field trials [40]. These and similar technologies are being developed in anophelines, some of which rely on CRISPR to achieve the desired outcomes, and will be discussed further below. With limited success of these technologies in species outside of *Anopheles stephensi,* alternative tools need to be developed for successful vector control in species such as *Anopheles gambiae, Anopheles coluzzii, Anopheles arabiensis,* and *Anopheles funestus* and the remainder of the malarial anopheline clade. If developed, these could represent a game-changing technological leap for these species.

## CRISPR—history and development

CRISPR was originally discovered in a variety of different systems [41–43], but its application for genetic engineering was the true epiphany. It was originally engineered into a minimal two-part system by Jennifer Doudna and Emmanuelle Charpentier, with CRISPR-associated protein 9 (Cas9) and a synthetically fused guide RNA (gRNA) to make genome edits in vitro [44]. It relies on a two-part system in which the Cas9 enzyme facilitates DNA cleavage, and it is targeted to a genomic target by the presence of a synthetic gRNA. This gRNA has a 20-base pair (bp) sequence which targets the genome via Watson–Crick base pairing, followed by a ~ 100-bp sequence that forms a secondary structure which complexes with Cas9. Bringing these two parts together enables targeted DNA cleavage of almost any sequence, so long as it has a PAM NGG protospacer motif. Different Cas systems can be used to target sequences with different PAMs such as Cas12a [45], though primarily canonical *Streptococcus pyogenes* Cas9 has been used in mosquitoes. CRISPR has truly revolutionized the genetic engineering of mosquitoes for basic biological study and vector control.

## Establishing the basic tools for CRISPR implementation: knock-ins, knock-outs, receptor-mediated ovary transduction of cargo and homology-assisted CRISPR knock-in

**Fig. 2 A–D:**
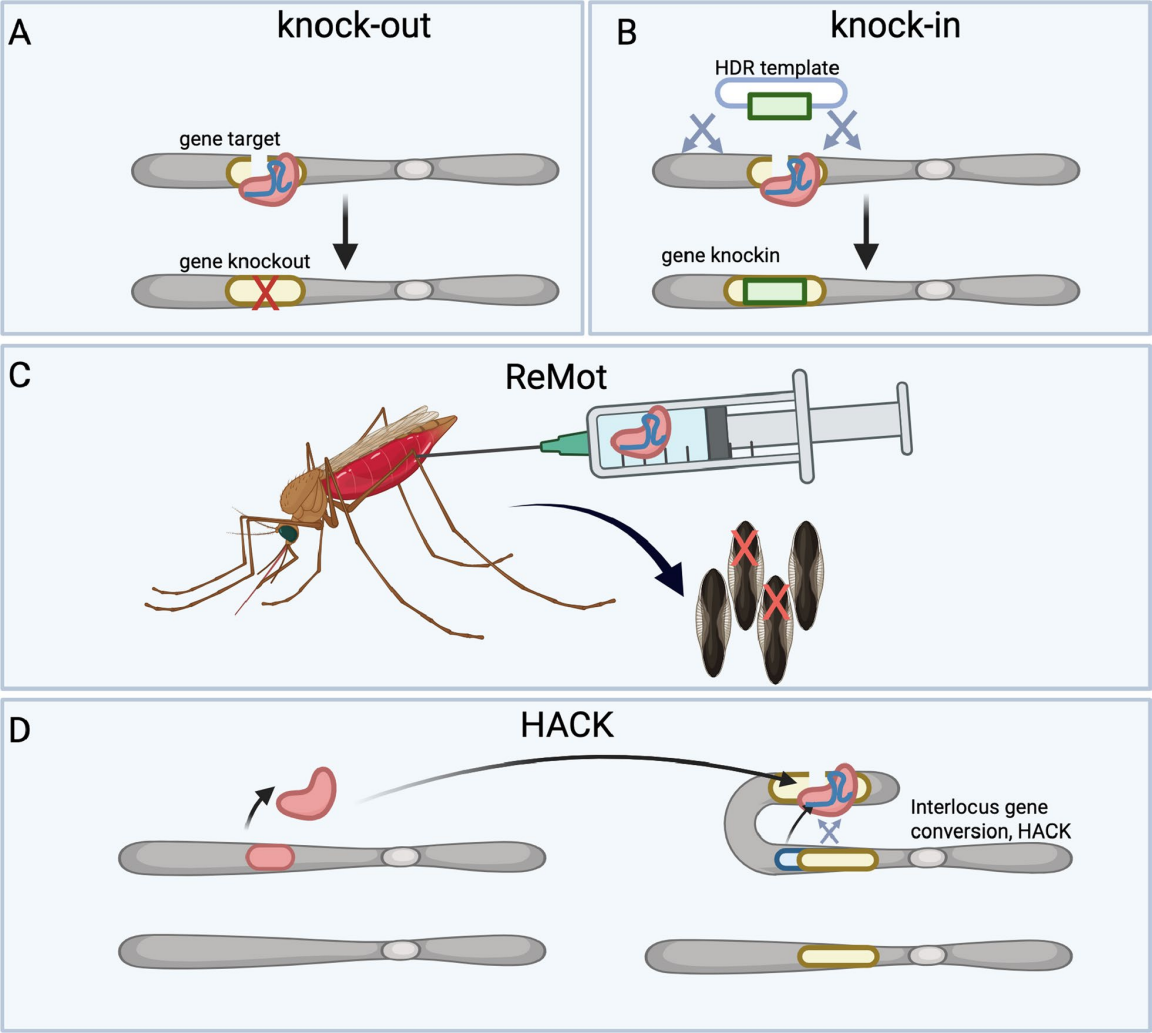
Basic tools for CRISPR implementation. **A** CRISPR-mediated gene knock-out to destroy gene function. **B** Knock-in using homology directed repair (HDR) to disrupt or augment the targeted gene. **C** Receptor-mediated ovary transduction of cargo (ReMot) injection of CRISPR components into the female hemolymph enables deposition of CRISPR components into the ovaries and subsequent mutagenesis of offspring. **D** Homology-assisted CRISPR knock-in (HACK)-based gene knock-in into the target locus from template transgenically expressed elsewhere on the chromosome. CRISPR-associated protein 9 (Cas9) and guide RNA (gRNA) are expressed* in trans*

Establishing the basic tools for CRISPR mutagenesis in *Anopheles* has followed on the heels of decades of research into embryonic microinjection and transgenesis protocols [46–50]. Zinc finger nucleases were used to make the first mosquito knock-out in *Aedes* [51], and TAL endonucleases (TALENs) were used to make the first knock-out anopheline [52], paving the way for CRISPR to be adapted in these species. The first demonstration of CRISPR in anophelines was the knock-out of fibrinogen-related protein 1 (FREP1) in *A. gambiae *[53] (Fig. 2A), followed soon thereafter by CRISPR-based gene drives, selfish genetic elements capable of spreading engineered traits through wild populations (discussed further below) [54, 55]. In *A. stephensi* and *A. gambiae* these works provided the first evidence of gene knock-in and knock-out, as well as outlining the basic CRISPR transgenesis toolkit, laying the groundwork for further research in similar organisms (Fig. 2B). Later, in lesser-studied anophelines, Li et al*.* [56] targeted the white eye gene in *Anopheles albimanus*, *Anopheles coluzzii*, and *Anopheles funestus* to prove this gene candidate was a good visual phenotypic marker, and provide evidence of the first CRISPR mutagenesis in these species. Also in *A. funestus*, another team proved gene knock-in was possible by again targeting the white eye gene for knock-in with a CFP-expressing cassette [57]. Together these works formed the basis of the basic molecular biological toolkit in these species.

Prior to 2018, all genetically modified (GM) mosquito lines were generated using the same basic paradigm of embryonic microinjection. Then in 2018 the traditional embryonic injection paradigm was turned on its head when Chaverra-Rodriguez et al*.* [58] invented ReMot, which allows for the delivery of CRISPR components to the embryo through injection of the mother (Fig. 2C). This work made generating knock-outs much more approachable and feasible for many labs that lack embryonic microinjection capabilities. Since its invention, it has been implemented in a wide variety of insect species, including *Anopheles stephensi* and *Anopheles sinensis* [59, 60], with promise to be easily adaptable to other anopheline species in the future. However, the technology has yet to be used to integrate transgenes, making it currently limited to the generation of CRISPR knock-outs. This work opened the door for other labs to engineer mosquitoes in the absence of traditional embryo microinjection facilities.

Gene knock-in based on CRISPR cleavage followed by homology directed repair (HDR) into the break is still a time-intensive process. Alternative CRISPR-based knock-in technologies have been developed, which promise to enable the development of ever more complicated gene knock-in lines. Notable among these is homology-assisted CRISPR knock-in (HACK) technology, which was developed by Lin and Potter [61] in *Drosophila* to enable gene knock-in. It enables knock-in, not from an exogenously provided homology template, but from a homology cassette inserted transgenically elsewhere in the genome. It then takes advantage of the rare, but naturally occurring, interlocus gene conversion phenomenon to knock-in the template into the target locus (Fig. 2D). It has been more recently used to develop neuronal knock-in lines in *A. coluzzii *[62], via a QF2 knock-in into the synaptic gene* bruchpilot*, proving its application for this species. Notably, HACK has also been used to generate gene drive docking lines in a haplolethal ribosomal and proteasomal locus in *A. gambiae* [63, 64]. This demonstrated the engineering of previously intractable loci and potentially facilitated the creation of a novel type of haplolethal-targeting gene drive [65]. Together HACK provides a novel option for gene knock-in of complex templates into otherwise genetically intractable loci, enabling ever more complicated gene drive designs (discussed further below).

## The study of anopheline basic biology to better understand the genetic basis of traits relevant for vectorial capacity

Understanding the basic biology of anopheline mosquitoes, and in particular the genes involved in traits relevant for vectorial capacity, can enable the development of novel vector control technologies targeting this pest. Using CRISPR to generate gene knock-outs and knock-ins has promised to revolutionize the study of targets involved in these important aspects of mosquito biology, and opened the door to the development of novel vector control strategies. Since the advent of CRISPR, many such genes have been targeted and studied. Here we will discuss a selection of them, including, but not limited to, *FREP1, LRIM1, Saglin, IR21a, SOA, msl-2, mosGILT*, and *ZPG*, among others. These knock-out lines have revolutionized the study of numerous topics, from host–pathogen interactions, to reproduction, to sex differentiation, to olfaction, to host-seeking, among many others.

## Studying the host–pathogen response

Using CRISPR to better understand the process of parasite development within the mosquito can inform the development of novel vector control strategies and be used to identify novel candidates for antimalarial gene drive cargoes. In the first demonstration of CRISPR in *A. gambiae*, *FREP1* was knocked-out (Fig. 2A**)**. FREP1 is a protein expressed in the midgut peritrophic matrix which binds traversing* Plasmodium* parasites, and knock-out was shown to reduce the number of developing parasites. This revealed that *FREP1* acts as a critical host factor that mediates* Plasmodium* invasion, and could be targeted as an anti-pathogen effector [66]. Similarly *LRIM1* is a mosquito immune response gene which plays a role in *Plasmodium* development in the midgut. It was originally targeted to generate a hyper-parasitic line for use in the production of the *Plasmodium falciparum* sporozoite protein vaccine PfSPZ. However, after using CRISPR to generate a knock-out (Fig. 2A), its role in microbiota regulation and reproduction was revealed instead, making it a suboptimal target [67]. Similarly involved in transmission of the parasite across the midgut is *mosGILT*. Following infection with *Plasmodium,* mosaic mutant individuals displayed significantly fewer oocysts, along with defects in ovarian development. Mutants displayed increased *TEP1*-mediated ookinete killing, and severely impaired vitellogenin production, which implicated this gene in the canonical *TEP1* complement-like response, making it a prime candidate as a antipathogen factor (Fig. 2A) [68]. Beyond the midgut, the salivary glands are another important site for study within the mosquito vector, as this is where sporozoites reside, waiting to be inoculated into a new host. Klug et al. [69] used CRISPR to develop fluorophore knock-in lines into three salivary gland genes, *AAPP*, *TRIO*, and *Saglin* to evaluate the blood meal-inducible properties of these promoters for use in GM vector control designs (Fig. 2B). In follow-up work they studied* Saglin* in more detail.* Saglin* was once thought to be the salivary gland receptor for the recognition of malaria sporozoites, but its CRISPR knock-out revealed instead that it is involved in parasite colonization of the midgut. Knock-out individuals harbored reduced parasite loads, implicating this gene in the host–pathogen response (Fig. 2B) [70]. In a work available on bioRxiv at the time of writing, Hoermann et al*.* [71] used CRISPR to generate mosquitoes overexpressing the antimalarial gene *REL2.* They did so within the midgut inducible gene zinc carboxypeptidase A1 in linked P2A-fusion manner. *REL2* is thought to act by activating factors essential for the mosquito immune response against the parasite, such as *Tep1*. Though transcriptional data indicated this gene was expressed, and there were associated fitness costs, the effect on the resultant parasite load was modest. Hoermann et al*.* [71] then generated a *REL2* knock-out and pursued a characterization of the knock-out phenotype, revealing its role as a regulator of the immune deficiency pathway (Fig. 2B). Some gene targets behaved as expected, while others did not, but overall the anti-pathogen effector toolkit available to researchers is expanding. All in all, we have only begun to scratch the surface of elucidating the molecular players involved in the mosquito-*Plasmodium* host–pathogen response, but CRISPR has enabled a robust start.

## Study of stimuli-seeking behavior

A variety of receptors involved in host- and oviposition-seeking have been targeted by CRISPR, revealing insights into these critical processes for disease transmission and reproduction. CRISPR knock-out of the ionotropic cooling receptor *IR21a* revealed that it drives heat-seeking and heat-stimulated feeding behavior, acting through cooling-mediated repulsion (Fig. 2A, B). However, mutant mosquitoes were still capable of blood-feeding, revealing a redundancy in sensory receptors critical for this trait [72]. Gene knock-out via knock-in of odorant receptor co-receptor (*Orco*) in *A. coluzzii* caused individuals to lack nearly all odor-evoked responses (Fig. 2B). They displayed defects in blood meal seeking, and in detecting oviposition sites, in addition to failure to detect Lindberger cheese, a well-established human odor proxy [73]. Similar results were obtained from* Orco* knock-outs in *A. sinensis* [74]. Also in the Asian mosquito *A. sinensis*, mutagenesis of the odorant binding protein AsOBP1 revealed decreased sensitivity to 1-tetradecanol and heptanal, two human chemoattractants [75] (Fig. 2B). Subsequently in *A. gambiae,* Laursen et al*.* [76] generated *Ir93a* mutants which failed to detect oviposition sites as well as close range attraction to a human hand, failing to probe when one was provided (Fig. 2B). Colocalizing with* Orco* is *Aclr76b*, an ionotropic receptor first studied in *A. coluzzii.* Interestingly, *Aclr76b* mutants display significantly increased responses to amines in some sensilla and decreased responses in others. Mutant females display insemination deficits and an inability to blood feed despite initially successfully host-seeking, implicating this receptor in both the olfactory and gustatory systems (Fig. 2B) [77]. In other work aiming to knock-out ammonium sensing, *Ye *et al. [78] generated CRISPR mutants in the ammonium receptor *AcAmt*, but surprisingly mutant mosquitoes still responded to ammonium and a variety of other odorants and instead showed significantly lower insemination rates during mating and increased mortality during eclosion (Fig. 2B). Previously uncharacterized in other Diptera, *MSAP* knock-out revealed it to be a novel molecular actor of odorant reception, discovered in *A. gambiae*, which promotes female mosquito attraction to human odor and enhances sensitivity to a variety of odorants (Fig. 2A) [79]. Then, in *A. coluzzii*, to study anopheline sensitivity to CO_2_, Liu et al*.* [80] used mutated gustatory receptors *Gr22, 23*, and *24*. Their work showed that *Gr23* and *Gr24* were required to maintain sensitivity to CO_2_, while *Gr22* mutants responded to CO_2_ at a weaker intensity. They proposed a model in which *Gr22* has a modulatory effect on the CO_2_ detection functionality on *Gr23/24* complexes, together further advancing our knowledge of detection of this critical host cue (Fig. 2B) [80]. Konopa et al. [62] used the CRISPR knock-in technology HACK to generate a T2A-QF2 knock-in in the neuronal gene* bruchpilot*, and used it to fluorescently label neurons. By comparing similar labeling of* Orco* mutant mosquitoes they were able to predict the extent of neurons expressing ionotropic receptors or other chemosensory receptors (Fig. 2D). Finally, Giraldo et al. [81] used CRISPR to insert the Q-system driver and responder elements to fluorescently label the chemoreceptor genes* If25a*,* Ir76b*,* Gr22* and* Orco*, for use in studying transcuticular calcium signaling (Fig. 2B). All in all, these works combine to form an increasingly robust toolkit for the study of different chemosensory processes in anopheline mosquitoes, potentially informing vector control strategies of the future.

## Study of reproduction

Targeting insect reproduction has been shown to be one of the best ways to control disease transmission, and understanding more about anopheline reproduction promises to enable development of novel vector control tools. Zero population growth (*Zpg)* germline-less CRISPR knock-outs have been used to demonstrate a few interesting principles important for our understanding of reproductive physiology, vectorial capacity and vector control. Firstly, *ZPG* knock-out females lack ovaries and are thus deficient in production of a critical insect hormone, 20E. These females helped reveal that *Plasmodium* development was intimately but not antagonistically linked to reproduction through a 20E-mediated mechanism, but allowed for faster parasite development, overturning dogma surrounding reproduction and infection competition (Fig. 2A) [82]. On the other hand, *ZPG* mutant males developed no testes, generating some of the first genetically sterile males in anophelines, helping to prove that genetic technologies could be used to generate sterile males for sterile insect technique (SIT; discussed further below) [83]. The study of reproductive processes in anophelines has informed the development of novel vector control tools, and upended our knowledge of host-parasite interactions.

## Study of sex differentiation

A better understanding of the biology of sex differentiation in anopheline species promises to enable the development of better sex-separation technologies for use in vector control in which male-only releases are desired. In *A. gambiae, *Valsecchi et al. [84] used binary CRISPR crosses to demonstrate that the gene *msl-2* plays a critical role in X-chromosome dosage compensation (Fig. 2A). More recently, Kalita et al. [85] similarly used CRISPR to identify and characterize a novel gene, *SOA* (sex chromosome activation), as a master regulator or for dosage compensation in *A. gambiae* (Fig. 2B). Also in 2023, following the discovery of the anopheline sex-differentiation gene femaleless [86], Smidler et al. [87] used CRISPR to generate an inducible system to robustly and reproducibly kill females. This resulted in a nearly pure genetic sexing system in *A. gambiae* [87]. This is a critical component for vector control systems, as releasing females is undesirable due to their ability to transmit disease (Fig. 2A). Fortunately, this technology could be ported into adjacent anopheline species in a fairly straightforward manner. Overall, CRISPR-based research into sex-differentiation pathways has yielded the fruitful production of actionable vector-control tools.

## SIT—historic vector control technologies

SIT is a proven method for the control of insect pests. Because the females of many insect species are monandrous (they only mate once), the mass release of sterile males causes population suppression. Therefore SIT releases act as a chemical-free, species-specific potent insecticide. SIT has been successfully implemented to control screwworm and medfly [88, 89] as well as *Aedes* mosquitoes [90], but releases have yet to be undertaken in anophelines. In *Aedes*, successful suppression of cases of dengue has even been achieved, demonstrating the potential of this technology to aid in disease eradication [14]. Traditionally, SIT males are generated by irradiation or chemical-based sterilization methods, but in anophelines this results in fitness costs that somewhat prevent males from being viable mates [91–94]. Furthermore, in other species, such as those of the genus *Aedes*, the required sex-separation is achieved via mechanical and robotic methods. However, historic methods for separating males from female anophelines have been limited to manual sorting at the pupal stage [26], or use of the now-banned neurotoxic chemical dieldrin, whose prohibition makes further use of this technology impossible [95]. Nonetheless, the proof of principle of SIT in anophelines has been shown using males treated with partially sterilizing doses of radiation [96], making the success of a SIT system using the precision of CRISPR an alluring possibility.

## Non-driving population-suppression vector control technologies

**Fig. 3 A–C:**
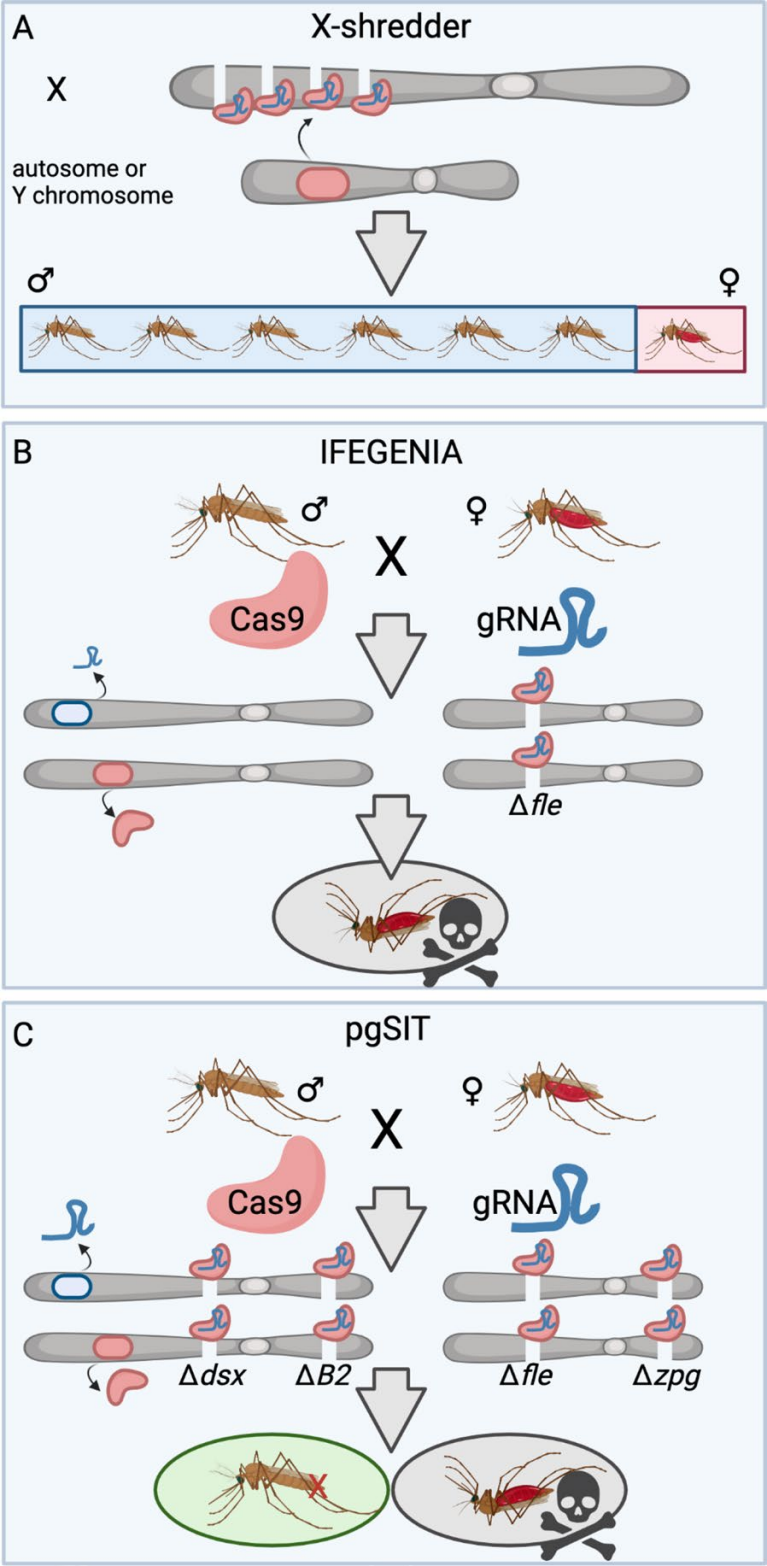
Non-driving CRISPR-based vector control technologies available at this time. **A** X-shredders encode CRISPR components from the Y chromosome set to target ribosomal repeats on the X chromosome. X chromosome shredding results in significant male bias. **B** Inherited female elimination by genetically encoded nucleases to interrupt alleles (IFEGENIA) technology involves crossing together Cas9 and gRNA lines to produce F1 offspring in which females are killed. It acts as a perfect genetic sexing strain and population suppression technology when males are released. **C** Precision-guided sterile insect technique (pgSIT) involves a similar Cas9 × gRNA crossing mechanism to that required for IFEGENIA, but additionally targets male sterilization gene targets and an additional female-incapacitating *dsx* gene target. It produces sterilized males en masse for release in SIT-like releases. For other abbreviations, see Fig. 2

Generating sterile males, or males whose offspring are incomparable with life, is considered critical for the development of SIT or adjacent SIT-like technologies for the practical suppression of wild vector populations. For these technologies, purely sterile or offspring-killing male populations need to be produced. Early efforts to generate sterile male populations were undertaken with the development of X-shredder technologies. Predating CRISPR, expression of the endonuclease I-PpoI in the testes via the spermatogenic* β2-tubulin* promoter enabled targeting of the ribosomal repeats on the X chromosome, causing it to be shredded. In some lines, prezygotic shredding of the X chromosome in the testes enabled the production of nearly pure (95%) male populations for use in vector control, while in other lines shredding the X-chromosome in the embryo from paternal deposition via sperm resulted in nearly pure (95%) embryonic lethality, leading to functionally sterile males [97, 98]. X-shredders are intended to be expressed from the Y chromosome, generating a selfish Y which biases its own inheritance, but can also be generated autosomally in a manner incapable of drive. This X-chromosome shredding technology was soon thereafter redeveloped using CRISPR autosomally, which was shown to have similar success [99] (Fig. 3A). While these lines were originally generated with the ultimate development of a Y-linked gene drive in mind [100], in the interim these lines were used in small scale field trials as a sterile-male proof of principle, which revealed important findings regarding the potential roll-out of GM mosquito technologies [101]. Yamamoto et al. [102], in another study aiming to develop sterile male *A. stephensi*, showed that pro-apoptotic effectors were expressed in the testes via the spermatogenic* β2-tubulin* promoter. Though this work did not utilize CRISPR, in was demonstrated that the transgenic males were 100% sterile, with no sperm observed in testicular tissue, and males could induce mating refractoriness in females following copulation [102]. All in all, these works add to the growing toolkit of sterilizing technologies available in anophelines for population suppression.

## Daughter-killing technologies

What these aforementioned technologies lacked is a system for phenotype induction; sterility in these lines was permanent and required constant addition of wild types to maintain the line, making them exceedingly difficult to scale. Homozygosing these lines was also an impossibility, a problem for rearing males at large scales. Developing a vector control tool in which sterility or offspring killing is truly inducible (but not leaky^3^) is of paramount importance if such a vector control technology is to be implemented at a continental scale. While RIDL and FsRIDL^4^ have been developed in *Aedes* and *Anopheles stephensi* [40], inducible vector control systems of this type have yet to be developed in other anophelines. Recently, a non-leaky inducible vector control system using CRISPR was developed in *A. gambiae* termed inherited female elimination by genetically encoded nucleases to interrupt alleles (IFEGENIA). By crossing together separate Cas9 and gRNA lines targeting the female-essential gene target *fle*, the authors were able to generate not only a nearly perfect genetic sexing system, but also a non-leaky inducible daughter-killing system akin to FsRIDL (Fig. 3B). In IFEGENIA, the pure F1 hybrid Cas9/gRNA transheterozygous male population is releasable, whose offspring then inherit mutant *fle* alleles as well as the mutagenic CRISPR-expressing transgenes. This results in profound daughter killing, making it a potential vector control system for scaled deployment and control. With *fle*’s conservation among anophelines, this work could be ported into adjacent species in a fairly straight-forward manner. This work presented the first scalable non-driving inducible vector control option for *A. gambiae* [87].

## Genetic SIT

In work that used a similar Cas9 × gRNA crossing scheme, as previously discussed above, Smidler et al. [83] targeted the germline development gene zero population growth (*zpg*) for mutagenesis. Though the females without ovaries generated therein were studied in Werling et al. [82] (discussed above), the males were found to be 95% sterile and were some of the first inducible sterile males generated in anophelines. In this work [83], the first evidence of population suppression using this type of bipartite transgenic CRISPR system in anophelines was presented. Combining genetic male sterilization and female incapacitation, precision-guided SIT (pgSIT) uses a similar non-leaky transgenic Cas9 × gRNA crossing scheme to inducibly produce populations of pure sterile males for wide-scale release in SIT-like campaigns (Fig. 3C). Importantly, this design allows for automatic genetic sterilization and female killing so eggs can be directly released into the environment, precluding any pupal or adult stage sorting steps. Originally developed in *A. aegypti*, *Drosophila* and *Drosophila suzukii* [103–105], it had yet to be developed in anophelines until recently. In a work which combined the sterilizing properties of *zpg-* and* β2-tubulin* targeting with the female-killing properties of *fle* mutagenesis, Apte et al. [106] developed a complete pgSIT system in *A. gambiae.* They demonstrated extremely robust female killing (> 99.9%), male sterilization (> 99.5%), and concurrent population suppression in cage trials. This system could be deployable using optical sex sorting of the F0 generation to establish the cross; however, fluorescent sex sorters could also be introduced into the Cas9 and gRNA lines to achieve maximal scalability via flow cytometry sorting. These works present novel male sterilization technologies that bring genetic SIT one step closer to field deployment.

## A brief introduction to gene drives

Population replacement, in which a disease susceptible population is replaced with a disease refractory one, has never before been attempted in mosquitoes in the wild. However, attempts at developing technologies to achieve this are underway. Notably, population replacement could be achieved using a technology termed gene drives. Gene drives are selfish genetic elements capable of biasing their inheritance to spread themselves and a desirable genetic cargo through wild mosquito populations. They promise to enable population replacement of disease-susceptible populations with refractory ones in addition to potentially causing prolonged population suppression. Notably, however, gene drives could also be used for population suppression by spreading sterilizing or androgenizing traits. They have been called a potential panacea [12], and show great potential to be a solution for malaria control.

## CRISPR-based gene drives for population replacement and suppression

Gene drives are selfish genetic elements that can bias or guarantee their own inheritance, allowing for different genetic engineering outcomes on a population-wide scale. One such type of gene drive being developed is a ‘homing drive’ which biases its inheritance through a homing-like mechanism, and is the primary drive type being developed in anophelines to date. When fertility genes are targeted by a drive, population suppression can result; however, when other more neutral genes are targeted, population replacement can be achieved. Specifically, ‘suppression’ indicates when the gene drive is able to make itself and the population be suppressed to a certain level or go extinct (albeit perhaps only temporarily). On the other hand, the term ‘replacement’ refers to the spread of the desired drive and genetic cargo to allelic fixation in the population. This effectively replaces the old unengineered population with a newly engineered one incapable of transmitting disease. Both types of gene drives have been in development over the past decade, and have been developed in multiple species, anophelines and other Diptera alike [65, 107].

A generic homing-based gene drive design encodes DNA cutting machinery transgenically from a specific locus. This cleavage machinery can be anything from a homing endonuclease (HEG) or CRISPR to modular nucleases such as TALENs or zinc finger nucleases. When repair of the dsDNA break occurs via HDR, the transgenic drive cassette is copied over into the break. If this occurs in the germline, germ cells are functionally converted from heterozygous to homozygous and are inherited in the next generation accordingly. This biases inheritance, causing a super-Mendelian (> 50%) inheritance pattern. This conversion of heterozygosity to homozygosity in the germline is what enables the drive to spread itself through a population over multiple generations. Importantly, homing drives ‘spread’ but this is not to be confused with the ‘spreading’ of a virus per se. The organism is either born with or born without a homing drive, it can not be ‘caught’ during the lifetime of the organism, unlike a virus. Furthermore, the copying/spreading only occurs functionally once per generation, so works best in those organisms with a short generation time.

## History of synthetic gene drives

When addressing the development of homing gene drives in anophelines, it is perhaps most straightforward to begin addressing them approximately chronologically and grouped by type. While the concept of using selfish genetic elements to engineer wild populations has been around for decades [108], the ability to engineer them in mosquitoes has been achieved much more recently. In a Herculean effort, the first proof-of-principle of homing drive in anophelines occurred before the advent of CRISPR, and was achieved by expression of the HEG I-SceI, set to target a transgenically inserted target sequence. This work [109] laid the groundwork to prove that artificial homing drives would be a viable strategy. However, without a naturally occurring I-SceI site in the genome of wild populations of mosquitoes, this technology would be forever limited to targeting synthetic loci in lab populations. Therefore, designing HEGs to target endogenous genomic sequences was of utmost interest. With the advent of TALENs came great interest in developing them into HEGs for homing drive purposes, and construction of multiple drives was attempted in multiple mosquito species around this time (personal correspondence). While TALENs were used to make the first knock-out anopheline [52], there is a notable absence of published literature on gene drives generated using TALENs, with the exception of a published drive in flies [110]. While one might suppose that research on TALEN-mediated homing drives was abandoned when CRISPR became available, the reality was that TALEN-expressing homing drives were likely prone to shuffling across the TALEN repeats, a phenomenon seen in other work [111], breaking the drives before appreciable data could be collected [110] (unpublished data and personal correspondence).

## Drive resistance

**Fig. 4 A–I:**
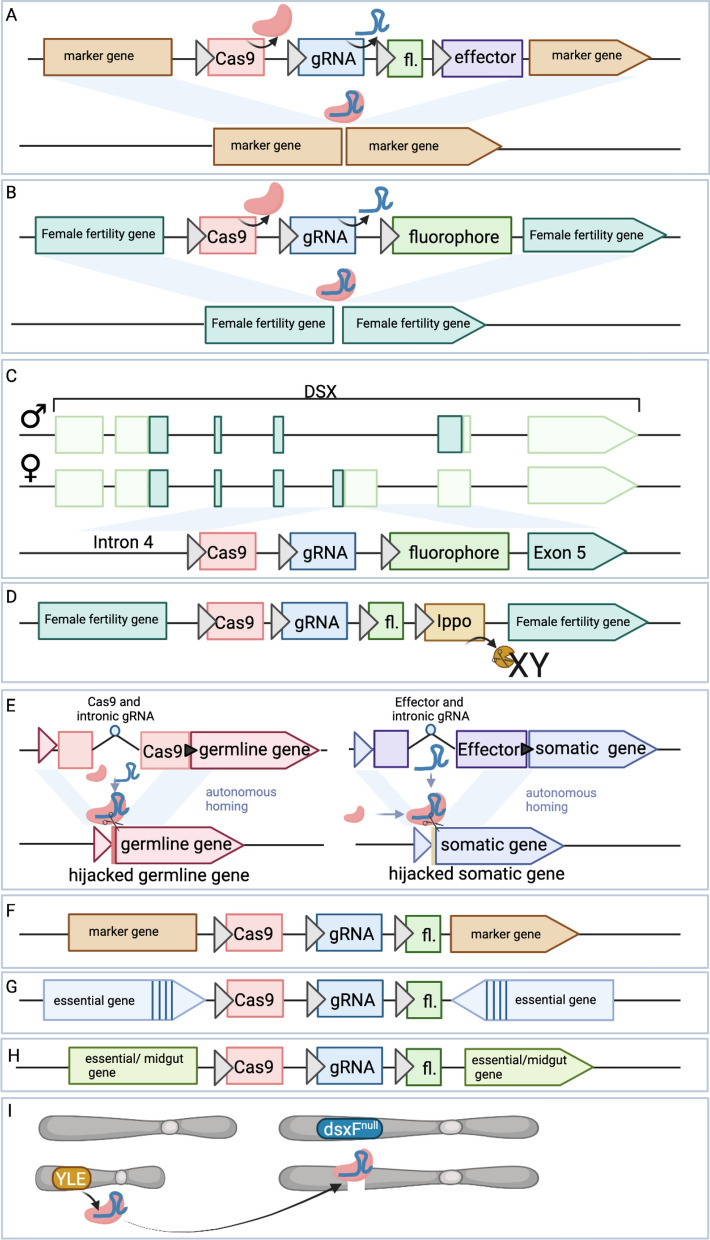
The general structure of gene drives described herein. Cas9 always shown in salmon pink, gRNAs in blue, fluorophore in green and effector gene in purple. Thick grey arrows indicate promoter sequences. **A** The first population replacement gene drive generated targeting kynurenine hydroxylase (*kh*), a visual marker gene that makes eyes white. Effector genes were included for antimalarial activity. **B** The first population suppression gene drives generated targeted three different female fertility genes for knock-out. Anti-pathogen effectors are not included in these designs as the drive function relies on gene knock-out for effect. **C** A *dsx-*targeting population suppression gene drive targets the female-specific intron4-exon5 boundary for knock-out. Knock-out causes female androgenization. **D** A sex distorter gene drive inserted in a female fertility gene expressing IppoI which targets the X chromosome in spermatogenesis. **E** Integral gene drives integrate Cas9 into an endogenous female fertility gene with the gRNA expressed from within an artificial intron. Effector genes are expressed from within endogenous somatic genes for midgut or salivary gland expression. The gRNA is expressed from within an artificial intron within the effector gene coding sequence. Cas9 is provided* in trans*. **F** A replacement gene drive harbored within the marker gene* Cardinal*. No anti-parasitic effectors are included in this design. **G** A population replacement gene drive within haplosufficient or haplolethal genes to minimize evolution of resistance. Essential genes are recoded to protect from cleavage (vertical bars in navy blue). **H** A essential or midgut gene disrupted by the gene drive causing gene knock-out. **I** A Y-linked editor (YLE) expresses CRISPR from on the Y chromosome, which causes cleavage and homing of a disruptive allele at the *dsx* locus. For other abbreviations, see Figs. 2 and 3

The first CRISPR homing drive in anophelines came with the development of a replacement drive targeting kynurenine hydroxylase (*kh*) in *A. stephensi*, which yielded a white eye phenotype when *kh* was disrupted (Fig. 4A). In this first work, the drive homed at an impressive 99.5% rate, but included antipathogen effectors that were not tested for their ability to inhibit parasite growth in the mosquito [54]. However, this drive demonstrated that the homing drive could rapidly fail to spread to fixation due to resistance [112], together providing some of the first evidence of homing drive resistance in anophelines. Resistance occurs when the drive-generated DNA break repairs not via HDR but via an alternative DNA repair pathway such as an end-joining pathway, resulting in a small mutation. These indels can no longer be recognized by CRISPR’s guide RNA, and if CRISPR can not cut, the drive can not drive—making these alleles resistant. In wild populations these small innocuous indels can theoretically outcompete the homing drive, forcing even the best-designed drives to extinction while the resistance allele could spread to fixation. These resistant alleles come in two types: R1 and R2 alleles. R1 alleles maintain the function of the gene, while R2 alleles disrupt the function of the gene. While both halt drive spread, R1 alleles pose the biggest risk to continual drive spread as they maintain the function of the gene while being resistant to the drive. Designing drives which circumvent these resistance alleles has become a major focus of the field.

## Population-suppression gene drives

The next demonstration of drive in anophelines was in *A.* *gambiae* in which three female fertility genes were targeted, with the aim of generating population suppression drives [55] (Fig. 4B). In this work, similarly high rates of homing and infertility of drive females were observed, demonstrating that population suppression homing drives can be developed in anophelines. However, the creation of resistant indel mutations that could theoretically inhibit drive spread making these drives suboptimal for release was also observed [55]. The authors characterized these resistant mutations and demonstrated how they could outcompete a drive to extinction [113]. Later, they aimed to build the best drive possible by testing different promoter elements for the regulation of Cas9 (*Zpg*, *nanos* and *Exuperantia*), and demonstrated that these regulatory sequences have a sizable effect on drive function [114]. They tested their preferred drive, regulated by *Zpg*, which showed drive up to 97% allele frequency but which ultimately was outcompeted by drive-resistant alleles. Later, they tested three of the aforementioned homing drives, in addition to a drive targeting *dsx*, and tested their ability to generate off-target mutations [115]. They identified 98 off-target sites, but found none contained more than four mismatches, for a maximum off-target frequency no higher than 1.42%. They observed that there was no accumulation of off-target mutations in a driving cage population and off-targets could be minimized if the drive was designed with a highly germline-specific promoter. They tested the ability of three of these fertility-targeting homing drives to home across the chromosome 2LA inversion and in different genetic backgrounds. They showed that drive was possible in all genetic backgrounds, demonstrating the robustness of homing drive across varied karyotypes [116]. Later, this group used these same homing drives to test the ability of an anti-drive to inhibit drive spread. They found that transgenic expression of the anti-Cas9 protein AcrIIA4 could inhibit full drive invasion in population cages, providing a new way to control gene drives [117, 118]. They analyzed the ability for these drives to convert drive-adjacent homology sequences during homing. They discovered that approximately 2/3 of homing events resolved within 50 bp of the cut site, with gene conversion over 400 bp being rare. Thus demonstrating that homing gene drives will likely have a minimal effect on diversity of surrounding sequences, and are unlikely to co-convert genes responsible for insecticide resistance for example [119] if such genes were to be adjacent to the drive. All in all, these fertility-targeting homing drives have revealed much about drive biology and have proven a valuable test case towards understanding gene drive function. While these drives are promising, the evolution of resistance alleles makes them suboptimal candidates for release at the current time.

Later this team developed a novel suppression homing drive, this time targeting the sex-differentiation gene doublesex (*dsx*), termed Ag(QFS1) (Fig. 4C). This drive targeted the highly conserved intron–exon boundary of the female-specific exon of *dsx*, generating non-viable intersex females, and suppressing the population as it spreads. High conservation of sequence at the splice site makes it theoretically less tolerant of resistance alleles. They demonstrated that this drive can spread to fixation, albeit with some exceptionally rare resistance alleles detected [120]. Overall, this has been the primary population suppression homing drive candidate developed to date, and it has undergone many follow-up studies to validate its performance. In progression towards field trials, this same group tested this drive’s ability to spread in large cage trials. In these cages they demonstrated its ability to crash the population as the drive spread to fixation, providing further evidence for this drive’s candidacy for field trials [121]. In a preprint on bioRxiv by Morianou et al., they develop a pipeline for discovering and testing drive resistance alleles generated by this Ag(QFS1) drive. In prior works this drive was found to generate few to no resistant alleles; however, in this work they discovered a number of resistance alleles and found that they can indeed inhibit drive invasion. They then generated a next-generation* dsx*-targeting drive which targets multiple sites simultaneously and demonstrated slightly improved performance parameters [122]. Overall, *dsx*-targeting drive designs present a promising population-suppression drive type for field application if resistance alleles from the selected drive remain rare which remains to be determined.

In an effort to construct a potentially more powerful population suppression homing drive, Simoni et al. [123] generated a sex-distorter gene drive by inserting male-biasing X-shredder components [98] into previously published population suppression drive designs (Fig. 4D) [55, 120]. In these drive designs, population suppression was modeled to be more robust, with male-bias coinciding with androgenization or a loss of female fertility, propelling the drive to fixation faster than other drive designs. X-poisoning systems have also been developed in *A. gambiae,* a tool inspired by X-shredder systems that target X-linked haplosufficient genes instead of the X-chromosomal repeats. When targeting haplosufficient genes, killing of X-inheriting daughter offspring results. This does not result in increased allele frequency, but does result in persistence of the nuclease element, producing a sex ratio distorter at the release frequency. In this work they targeted three X-linked ribosome protein genes for cleavage via autosomal-based transgenic lines and demonstrated robust proof-of-concept of postzygotic lethality of female progeny, demonstrating that this type of element can be used for male-biased sex ratio distortion in *A. gambiae* [124], an important feature for GM vector control systems.

A selfish Y-chromosomal element which biases its own inheritance but does not drive by traditional HDR is termed a Y-linked editor (YLE) (Fig. 4I). In one example it is composed of CRISPR elements targeting *dsx* encoded on the Y-chromosome, to generate androgenized females dominantly. It removes females from the population, thereby maintaining the YLE at approximately the same population frequency as it was released. This work presents a novel type of vector control tool for anophelines that is theoretically better than SIT, X-shredder, or RIDL-like technologies [125] due to its drive like properties. It presents a unique intermediate between non-driving technologies such as pgSIT, X-shredder, RIDL, and formal homing drive technologies.

In another work available on bioRxiv at the time of writing this work, Xu et al. [126] set out to develop the first population suppression gene drive in *A. stephensi.* They generated a *dsx-*targeting gene drive targeting the female-specific exon of *dsx* with nanos-Cas9, but which was recessive sterile in both males and females making population invasion impossible (Fig. 4C). They discovered boosted homing when crossed to Vasa-Cas9 but also some resistance alleles at low frequency, thus demonstrating that such a system in *A. stephensi* may be possible. They suggest that this technology may enable faster suppression than SIT and RIDL technological alternatives [126] for this species; however, with the significant evolution of resistance, we suggest that this technology may require further development before releases are undertaken.

## Population-replacement gene drives

To enable population replacement homing drives which inhibit resistance, integral gene drives (IGDs) were developed (Fig. 4E) [127, 128]. Modeled first then constructed [127], these drives are inserted within endogenous essential fertility or somatic genes, making any resistance alleles which do arise highly deleterious. They are inserted in such a way that allows for proper coding of the endogenous gene as well as in-frame Cas9 separated by a ribosome skipping signal, T2A or P2A, with the gRNAs and selectable marker inserted in a synthetic intron therein. Regulated by the endogenous promoter sequence, Cas9 is inserted within germline endogenous genes while antipathogen effectors are inserted within midgut or salivary gland genes for appropriate expression. Antipathogen effectors are driven* in trans* by the activity of Cas9 and gRNAs from their distal locus. Non-HDR-based repair of the DNA break at the drive target locus would disrupt the female fertility or midgut/salivary gland gene making most mutations highly deleterious. However, correct homing would copy over the Cas9 and gRNA cassette or effector, together making a powerful population replacement drive strategy. Elsewhere, the authors outlined the development of the previously proposed IGDs, by inserting antipathogen effectors within three different endogenous midgut-expressing genes and assaying for different levels of parasite development. While they demonstrated little to no effect on parasite infection intensity within the midgut, they did demonstrate biased inheritance of the drive element when crossed to a source of Cas9 [128]. They also observed a significant accumulation of resistance alleles, suggesting that these drive designs may not be as good at hindering resistance as previously thought. This team later developed *zpg*-targeting IGDs that were intended to be fertile by design but were, however, dominantly sterile in females due to germline mutagenic activity. They demonstrated biased inheritance in males and the ability to drive Scorpine-expressing nonautonomous effector IGD cassettes in cage trials despite the *zpg*-targeting IGD drive spreading towards extinction [129]. In another work by this group, they used an IGD-like design to augment midgut expressing genes to express two antimicrobial peptides melittin and magainin2. These nonautonomous effector designs are intended to be driven by an IGD design providing Cas9* in trans*. They demonstrated that each of these lines was able to slow sporogonic development, having a likely effect on malaria transmission [130]. All in all, while IGDs are theoretically promising, their practical application might be hindered by the evolution of resistance at the cut site, making them imperfect for consideration for field trials at this time.

In an effort to generate a working population replacement drive in *A. gambiae* Carballar-Lejarazu et al. [131] inserted a drive design into the* Cardinal* (*cd*) locus (Fig. 4F). Though these drive designs did not include anti-pathogen effectors, they were able to demonstrate successful drive invasion in cage trials; however, some resistant mutant individuals were identified that may inhibit drive in subsequent generations. The authors purport that this drive design fits the target product profile for a releasable drive in this species. Although they largely did not observe resistance alleles in caged populations of one of their homing drive lines, AgNosCd-1, they decided to examine the generation of resistant alleles more closely. They observed significant resistance allele generation specifically in the maternal lineage of drive inheritance due to embryonic cleavage and nonHDR repair of the paternal allele, potentially capable of inhibiting drive spread [132]. They developed two complete population replacement drive designs in* Cardinal*-carrying antipathogen effectors and tested them in *A. gambiae* and *A. coluzzii* (Fig. 4A). There they observed significant drive invasion in caged populations, but some drives were outcompeted by resistant alleles, and while parasite loads were reduced, they were not eliminated. This presents the most advanced replacement drive developed in anophelines to date, and the authors suggest it meets the target product profile for a replacement drive in these species [133]. However, we argue that anti-malarial effectors need to have a stronger anti-pathogen effect and drives need to be less prone to resistance before stating that these designs are ready for field trials.

In another design type, essential genes can be recoded carrying CRISPR drive components adjacent to make most non-drive mutant outcomes lethal (Fig. 4G) [65]. Recoding is achieved by changing the codon but not the amino acid coded for, thereby altering the targetable DNA but not the coded protein. Cleavage occurs in the non-recoded wild type allele, with recoding protecting the drive-containing chromosome from cleavage. The Cas9 and gRNA drive components are inserted transgenically adjacent to, but outside, the recoding. When homing occurs, the drive and recoding are carried over into the break, and if homing does not occur then mutations in the essential gene eliminate that allele from the population. In one such design in *A. stephensi*, the haplosufficient essential gene kynurenine hydroxylase was targeted [134]. In this work the drive achieved 95% fixation in cage populations, with the remaining presumably being drive-resistant individuals, and some loss due to lethal mosaicism.^5^ Because the targeted gene was haplosufficient, some resistant alleles could be tolerated in heterozygous form, making this an imperfect design to reach complete population replacement, though > 95% drive remains impressive. In theory, such a design in a haplolethal gene (loss of one copy is lethal) [135] should enable a much stronger drive design capable of unilateral population replacement; however, no such drive design has yet been published in anophelines, but was attempted in Smidler et al. [63, 64], although their construction might be theoretically quite difficult due to their haplolethality.

In another haplosufficient essential-targeting drive design, albeit without any recoding, the authors Fuchs et al. [136] developed a drive targeting a highly conserved gene analogous to *Drosophila* Smrter (*Smr)* in an attempt to limit the evolution of resistance alleles (Fig. 4H). The drive was designed to be homozygous inviable making it capable of population suppression as it spreads; however, a sizable number of resistance alleles were isolated, again suggesting that targeting haplosufficient genes may not be the best course of action for the building of robust replacement drives.

In work by Green et al., the lipid transport protein lipophorin was hijacked to express an anti-malarial antibody Sc2A10, and a marked reduction in parasite load was demonstrated from this transgenic (Fig. 4H). They tested multiple possible drive designs to drive this transgene* in trans* through the population, with one concurrently homing through and disrupting the pro-parasitic gene* Saglin*, a top candidate. While they did observe the drive driving to high levels, they did, however, observe resistance alleles and instability in the multi-gRNA array. Together, these authors presented a novel strategy for an anti-malarial replacement drive [111], but one which is not ready for field trials.

Though not under development yet in *Anopheles*, their imminent development warrants a brief mention of Cas12a nuclease gene drives. In *Drosophila,* under development are gene drive systems using temperature sensitive Cas12 nuclease [137]. In this latter work, they demonstrated that gene drive function could be modulated by temperature, i.e. inactive at low temperatures and active at high temperatures. Further, by combining them with Cas9, they could develop ‘double drives’ using both nucleasaes together. These types of systems are likely to be developed in anophelines imminently and present an exciting avenue of future research.

## Drive-supporting research

In a work intended to support the development of gene drives, Terradas et al. performed in situ hybridization staining of putative germline-specific Cas9 transcripts in two different homing drive lines in *A. stephensi*. In this work nanos-Cas9-expressing and Vasa-Cas9-expressing driver lines were assayed. They observed different localization patterns for each drive in female germline tissues consistent with the different activities of each drive. They also evaluated the expression profiles of *spo11*, *oskar*, *snail*, *zpg* and *β2-tubulin* for their potential candidacy as germline regulatory elements for Cas9 in gene drives, together improving the toolkit available for constructing gene drives.

## Selecting a GM vector control tool

**Fig. 5:**
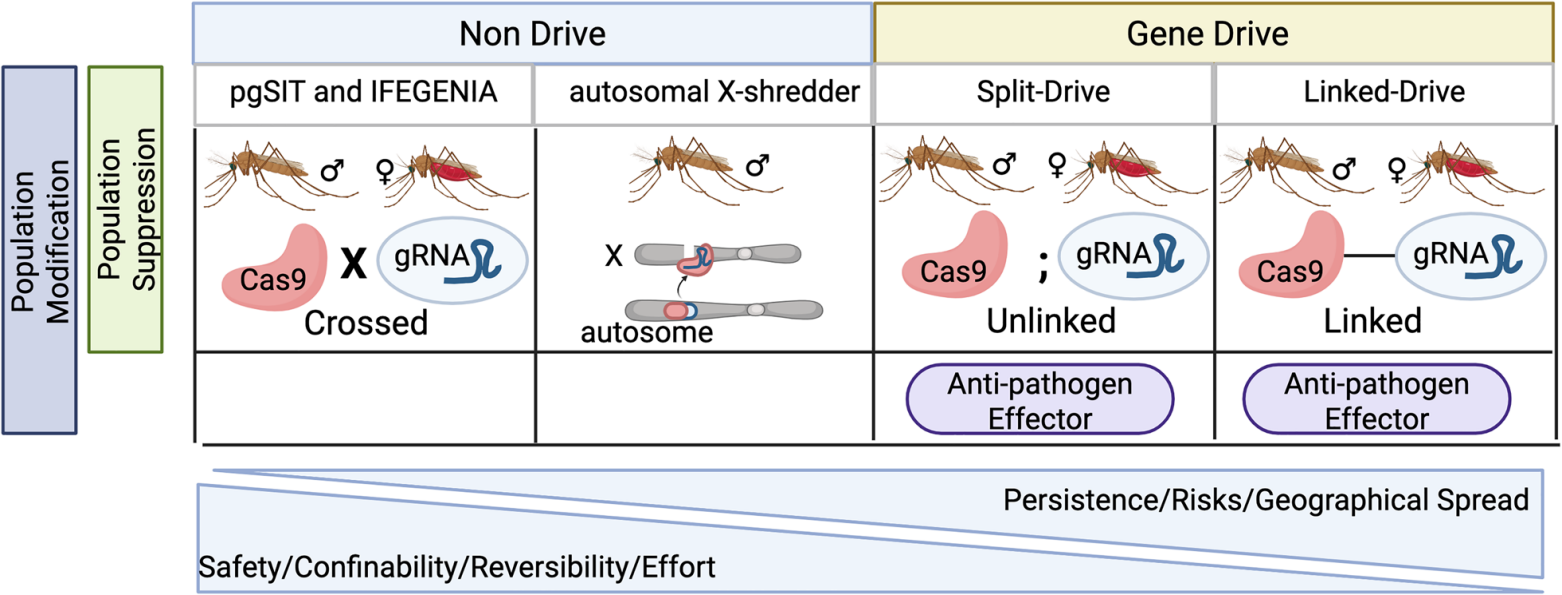
Population suppression and modification vector control technologies have different risks associated with their release and possible subsequent spread

All in all, while *dsx*-targeting population suppression drives seem more mature and ready for extended field trials [120], all replacement drives to date leave something to be desired either due to suboptimal anti-pathogen effector function or evolution of drive resistance. And while some gene drives may be ready for further trials, by nature of their ability to spread, a single release should not be undertaken lightly as it could result in modification or elimination of anophelines on a continental wide scale. Therefore, while they have been developed molecularly, the process of seeking regulatory approval for their release is still ongoing. Non-drive vector control strategies such as IFEGENIA, pgSIT and Split drives may be better alternatives (Fig. 5). Split drives which separate Cas9 and gRNA components have not yet been extensively developed in anophelines but could represent a more controllable intermediate between full drive and non-driving technologies. With this increased control comes a lower risk, easier regulatory approval, and better safety parameters.

## Conclusions

All in all, CRISPR has revolutionized many aspects of biology. In particular, it has upended both the study of traits implicated in the vectorial capacity of malarial anopheline mosquitoes and the development of next-generation vector control strategies. It has revolutionized our understanding of neurology, olfaction, reproduction, oviposition, chemosensation and much more. It has also enabled the development of an ever expanding toolkit of GM vector control strategies including, but not limited to, pgSIT, IFEGENIA, and X-shredders. It has also facilitated the development of a novel class of designer selfish genetic elements, termed gene or homing drives, which, if approved for use and proven effective, could revolutionize the application of vector control. It has upended the control of deadly diseases such as malaria, providing the foundation for multiple new vector control tools for the species that spread them. It has also informed progress in the study of other species of Diptera, enabling advancement beyond just the genus* Anopheles*. In many cases, the vector control tool has been almost fully developed, and it is almost time for regulators to proceed with the selection of which technologies will be implemented.

However, despite all the progress to date, some work still needs to be done to finalize the application of CRISPR-based vector control tools. Technologies such as IFEGENIA and pgSIT still require the inclusion of a fluorescent sex-sorting system, such as SEPARATOR, to maximize scalability [138]. X-shredders similarly require redesign to be inducible to allow for release programs to scale. Gene drives, particularly the *dsx*-targeting suppression drive, require regulatory approval for field release; however, drive resistance may become an issue. And while population replacement drives have been developed, the anti-malarial effectors need to be more effective before releases should be undertaken. The evolution of resistance alleles should be closely monitored in both types of drives to guarantee robust drive over time. All in all, practical vector solutions for direct application in affected areas are nearing completion and are almost ready for deployment.

## Data Availability

Data supporting the main conclusions of this study are included in the manuscript.

## Abbreviations

AAPP: Anopheline antiplatelet protein
AcAMT: *Anopheles coluzzii* ammonium transporter
AcIr76b: *Anopheles coluzzii* ionotropic coreceptor 76b
AgNosCd-1: An* Anopheles gambiae* gene drive
Ag(QFS1): An* Anopheles gambiae* gene drive
AsOBP1: *Anopheles stephensi* odorant binding protein 1
Cas9: CRISPR-associated protein 9
CI: Cytoplasmic incompatibility
CRISPR: Clustered regularly interspaced short palindromic repeats
DNA: Deoxyribonucleic acid
Dsx: Doublesex
Fle: Femaleless
FREP1: Fibrinogen-related protein 1
FsRIDL: Female-specific release of insects carrying dominant lethal
GM: Genetic modification/genetically modified
GR22, 23, 24: Gustatory receptor 22, 23, 24
gRNA: Guide RNA
HACK: Homology-assisted CRISPR knock-in
HDR: Homology-directed repair
HEG: Homing endonuclease
IF25a: Interferon regulatory factor 25a
IR76b: Ionotropic receptor 76b
IFEGENIA: Inherited female elimination by genetically encoded nucleases to interrupt alleles
IGD: Integral gene drive
IIT: Incompatible insect technique
IR21a: Ionotropic receptor 21a
IR93a: Ionotropic receptor 93a
I-SceI: Restriction enzyme ISceI
kh: Kynurenine hydroxylase
LRIM1: Leucine-rich protein 1
MosGILT: Mosquito gamma-interferon-inducible lysosomal thiol reductase-like protein
msl-2: Male-specific lethal 2
Orco: Odorant receptor co-receptor
pgSIT: Precision-guided sterile insect technique
R1, R2: Resistance alleles
REL2: Relish 2
ReMot: Receptor-mediated ovary transduction of cargo
RIDL: Release of insects carrying dominant lethal
SEPARATOR: Sexing element produced by alternative RNA-splicing of a transgenic observable reporter
SIT: Sterile insect technique
Smr: Smarter
SOA: Sex chromosome activation
TALEN: TAL endonuclease
TEP1: Thioester-containing protein
YLE: Y-linked editor
Zpg: Zero population growth
20E: 20 Hydroxyecdysone

## Footnotes

1 It is obvious that there must have been some ecological impact given the nature of the interventions; however, we failed to identify any formal studies that were conducted on this topic at the time. Whatever the ecological ramifications of mosquito elimination were, they were either undone in the intervening decades or were sufficiently insignificant to not warrant a cautionary tale. This is with the exception of the ecological effect of the chemical DTT, as summarized in the book* Silent Spring*, which caused significant harm to avian wildlife [13]. However, any ecological effect of mosquito population suppression itself was unnoted [13].

2 The ethics of disease eradication are occasionally raised during discussions surrounding malaria eradication. Notably, collectively humanity decided that smallpox was worthy of eradication due to its gruesome effects on humankind, and similarly few object to the imminent eradication of the Guinea worm, suggesting that the eradication of malaria—which disproportionately affects the world’s poor—should be granted the same approval.

3 ‘Leaky’ refers to the penetrance of the phenotype. In RIDL and fsRIDL their phenotypes are incompletely controlled by the addition of tetracycline making the system leaky. Non leaky systems are those in which the phenotype can be induced purely genetically, resulting in no aberrancy in the phenotype.

4 FsRIDL is similar to RIDL in that it relies on a chemically repressible lethal transgene. However, FsRIDL expresses specifically within the female flight muscles, resulting in males which sire daughters incapable of flight when in the absence of the repression chemicals, tetracycline or doxycycline.

5 Lethal mosaicism is when offspring from a drive individual experience lethality due to mosaic mutagenesis of the target sequence.
